# The relationship between social frailty and loneliness in community-dwelling older adults: a cross-sectional study

**DOI:** 10.1186/s12877-024-04666-2

**Published:** 2024-01-18

**Authors:** Zhixiao Li, Jinjin Gu, Peiling Li, Jiaqi Hu, Shanshan Wang, Panpan Wang, Lin Zhou, Yi Yun, Yan Shi, Peng Wang

**Affiliations:** 1 School of Nursing and Health, Zhengzhou University, Zhengzhou, China; 2 School of Basic Medical Sciences, Zhengzhou University, Zhengzhou, China; 3 Tongji Medical College of Huazhong University of Science and Technology, Wuhan, China; 4 College of Public Health, Zhengzhou University, Zhengzhou, China; 5 School of Nursing, the Hong Kong Polytechnic University, HongKong, China; 6 Henan Electric Power Hospital, Zhengzhou, China

**Keywords:** Community-dwelling older adults, Social frailty, Loneliness, Convoy model, Influencing factors, Correlation studies

## Abstract

**Background:**

Social frailty (SF) is associated with multiple adverse health outcomes, yet there has been an inadequate focus on social frailty. The convoy model portrays the social networks through the perspective of the life course, thus providing a framework to explain the occurrence of social frailty. This study aimd to figure out the prevalence of social frailty and loneliness among community-dwelling older adults and to explore their correlations based on convoy model.

**Methods:**

This was a cross-sectional study, and 295 older adults from 10 communities of Zhengzhou in Henan Province participated in the study. Social frailty and loneliness were assessed separately with the Social Frailty Scale and University of California at Los Angeles-Loneliness Scale. The scores of social frailty of the older adults in different characteristic communities were compared by independent sample t-test and single factor analysis of variance. The influencing factors of social frailty were analysed by multiple stepwise linear regression and the structural equation model. The correlation between social frailty and loneliness was analysed by Pearson correlation analysis.

**Results:**

The total scores of social frailty and loneliness of the older adults in the community were (2.09 ± 1.53) and (43.19 ± 8.91), respectively. There was a moderate positive correlation between social frailty and loneliness (*r* = 0.621, *P* < 0.01). The results of multiple stepwise linear regression analysis showed that age, living styles, balance of payments, and loneliness were the main influencing factors of the social frailty of older adults in the community (F = 27.180, *P* < 0.001). The structural equation model of social frailty fitted well (χ^2^ = 47.292, df = 26, χ^2^/df = 1.819, *P* = 0.007; RMSEA = 0.053, 95%CI (0.028, 0.076), *P* = 0.359; GFI = 0.971; AGFI = 0.939; NFI = 0.904; IFI = 0.955; TLI = 0.918; CFI = 0.953; SRMR = 0.0466).

**Conclusions:**

The convoy model had certain applicability in explanation of the relationship between loneliness and social frailty among older adults in community. The incidence of social frailty among the older adults in the community was high, and loneliness was at a medium level. It is necessary to strengthen the intervention of social frailty and loneliness of the older adults in the community, improve the quality of life of the older adults, and promote the development of healthy aging.

## Background

Frailty includes four aspects which are physical frailty, psychological frailty, social frailty, and environmental frailty. Social frailty is an important dimension of frailty, which means that individuals continue to lose one or more important resources to meet their basic social needs [1, 2]. The classic definition of social frailty refers to an individual's vulnerability or risk of decline in their social functioning, characterized by a lack of social resources, weak social networks, and low levels of social engagement [3]. These factors can lead to social isolation, loneliness, and loss of independence. As per this definition, social frailty is not just a functional, but also a social problem, which has a significant impact on the overall quality of life of older adults. With the gradual deepening of China's aging degree, the social participation, family support, and economic status of the older adults are seriously inadequate, which can easily lead to the occurrence of social frailty in the older adults [4, 5]. The middle-aged and older adults in China were at a moderate level of frailty, and the degree of social frailty was the most prominent [6]. Social frailty of the older adults occurs before physical frailty and then accelerates the process of physical frailty, which is related to various adverse health outcomes (e.g., falls, hospitalization, disability, death, etc.) and has certain predictability for death in related studies [7–9]. With populations worldwide continuing to age and life expectancy on the rise, the upward trend of social frailty has become an increasingly pressing issue. This has resulted in a growing number of older adults facing the risk of social frailty, which can have profound implications on healthcare systems and society [10]. Addressing social frailty has therefore become paramount in ensuring that the older adults maintain their health in their later years. However, the current domestic researches on the frailty of the older adults mostly focus on the physiological level, while ignoring the social level of frailty.

Loneliness is an unpleasant emotional experience, arising from the decrease in quality and quantity of individuals’ social relationships [11]. It is a subjective state of social isolation, accompanied by a lack of intimacy and narrow social networks [12]. The high incidence of loneliness among older adults seriously affects the subjective well-being and physical and mental health of older adults [12, 13]. Some studies have pointed out that loneliness is closely related to social frailty [14–16]. However, there is a lack of research on the relationship between social frailty and loneliness of the older adults in the community.

The convoy model presents a perspective that social relationships vary for persons depending on various personal and situational characteristics [17] and shows the factors of multiple dimensions (i.e., structure, social support, and support satisfaction) from social relationships within a life span or life course framework. All of the above finally contribute to the well-being, health, and quality of life of individuals [18]. The model gives an explanation that loneliness results from the narrow scope of social networks and lower frequency of communication [19]. At present, little was known about social support (the aid, affect, or affirmation people exchange) or specific factors that might predict the support an individual was likely to need or receive [17]. While the convoy model proposed that social relations could be predicted on the basis of specific, identifiable, antecedent factors such as personal and situational characteristics [20]. In the research, we defined loneliness as a state of absence of emotional support from convoy support, and social frailty was a variable of convoy quality.

**Fig. 1:**
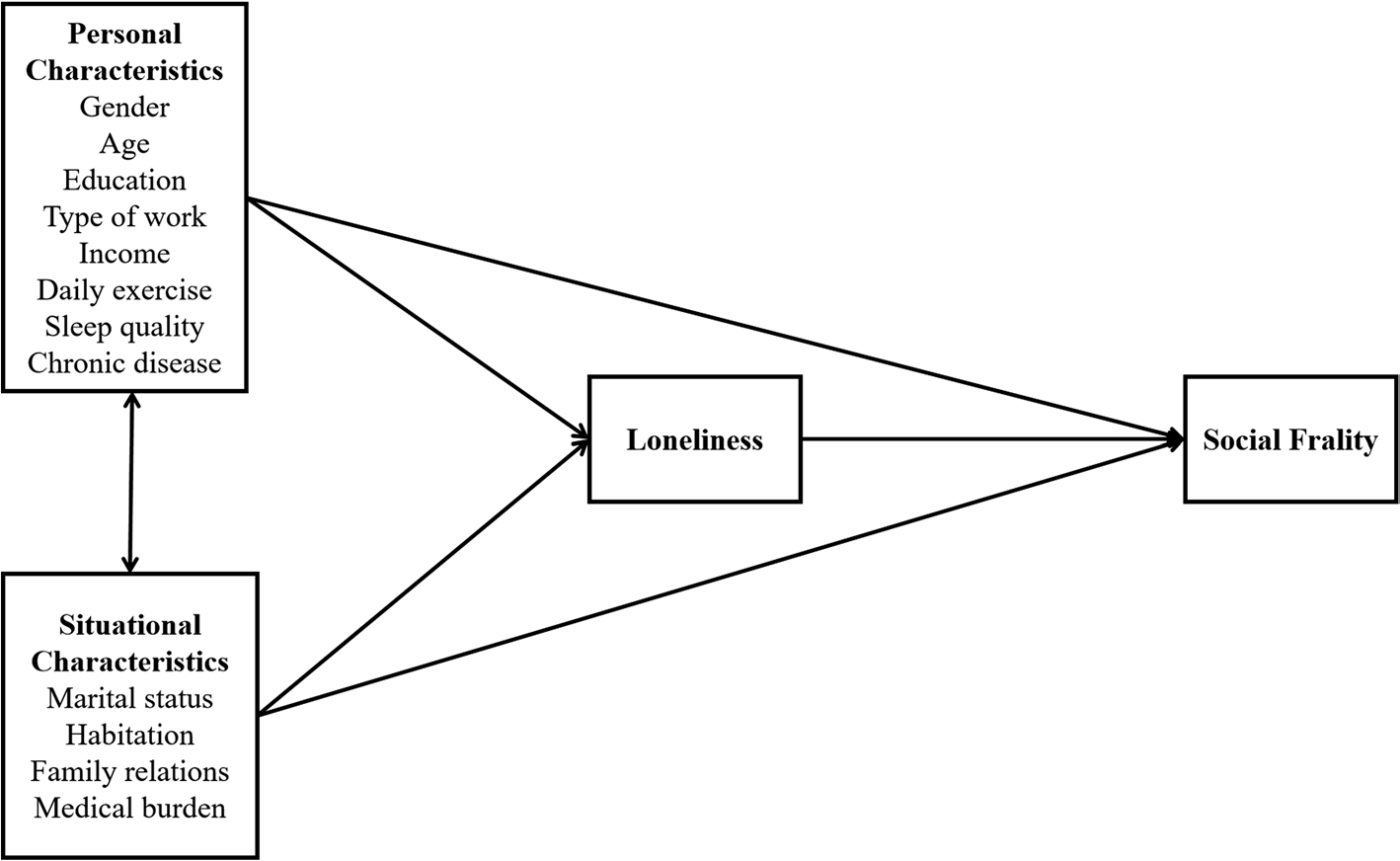
The model assumptions for social frailty among older adults in the community

Older adults tend to focus their attention primarily on close family members and the emotional components of social interactions. So, with the lack of social resources, shrinking of social networks, and less social engagement, they are more likely to suffer social frailty. The convoy model provides a comprehensive approach to reducing loneliness among older adults. But it has no sound evidence to prove the relation between social frailty and loneliness. Therefore, building on the convoy model and literature review, a preliminary theoretical model was formulated to explore the factors of social frailty in older adults, as well as the relationship between social frailty and loneliness (Fig. 1). In this model, we proposed four elements: personal characteristics, situational characteristics, loneliness, and social frailty. Personal characteristics included individual factors such as age, education, type of work, income, sleep conditions, gender, and so on. Situational characteristics included marital status, habitation, family relations, medical burden, and so on. The theoretical model built on individual and situational characteristics to allow for a more in-depth assessment of the factors that determine and influence social frailty. Loneliness was a result of the narrow scope of social networks and lower frequency of communication and could be affected by individual and situational characteristics. And loneliness as a state of lack of emotional support might further influence the outcome of social frailty. Therefore, we suggested the assumption that loneliness was associated with social frailty.

This article aimd to investigate the current situation of social frailty and loneliness of community-dwelling older adults in China. Additionally, we intended to preliminary explore the factors of social frailty and the correlation between loneliness and social frailty. Most importantly, this study sought to bring public awareness to social frailty and mental health issues among older adults in the community, offering a theoretical foundation for implementing interventions and fostering the development of healthy aging in the future.

## Methods

### Study design and setting

The study utilized a cross-sectional design, and was approved by the Ethics Committee of Zhengzhou University (ZZUIRB2022-59). The survey was conducted from May to October 2022 in 10 communities which had close co-operation with our research team, from Zhengzhou City, Henan Province, China.

### Sampling and participants

Convenience sampling and snowball sampling were selected in all communities to recruit older adults for the survey study because the COVID-19 pandemic made it impossible to use probability sampling. In order to achieve sufficient power, we conducted a priori power analysis to calculate how many participants would be needed. It was calculated that 262 participants would be suitable, assuming a medium efect size of 0.2, power of 0.9 and alpha of 0.05. Initially, approximately 350 potential respondents were contacted to participate in the survey, of which 316 expressed interest in participating. From those interested, they all respondents successfully completed the survey. After rigorous data screening and cleaning, a total of 295 older adults were included in the final analysis. Inclusion criteria: 1) age ≥ 60 years; 2) living in the community for more than 1 year; 3) clear consciousness and certain reading level; 4) informed consent and voluntary participation in this survey. Exclusion criteria: 1) those with mental illness or history of mental illness; 2) those with communication impairment.

### Data collection

Date was collected in person from individuals who met the inclusion criteria from May to October 2022, using a questionnaire based on the premise of obtaining informed consent from the respondents. We utilized a combination of self-administered questionnaires and face-to-face interviews for data collection. Participants were provided with the option to complete the questionnaire independently or receive assistance from our team through face-to-face interactions. The face-to-face component involved verbal communication, and our team members assisted respondents in filling out the forms during in-person interviews. After the deletion of questionnaires containing missing values and with low-quality, 295 valid questionnaires were used in the analysis, with a valid response rate of 93.35%.

### Measures

#### General social demographic and clinical characteristics questionnaire

This part was designed with reference to relevant research, mainly including the gender, age, educational level, habitation, type of work, family relations, marital status, daily exercise, sleep quality, the number of chronic diseases, personal income, and medical burden.

#### The social frailty scale

The Social Frailty Scale (Help, Participation, Loneliness, Financial, Talk Scale, HALFT) was developed in 2018 based on the longitudinal cohort study of aging in Beijing from 2004 to 2012 [7]. The inability to help others, limited social participation, loneliness, financial difficulty, and reduced social communication were used to evaluate whether the respondents were in a state of social frailty. The answers to each question were divided into ' yes ' or ' no ', and the answer to ' no ' was 1. The total score was 5 points. 0 points meant no social frailty, 1–2 points meant pre-social frailty, and ≥ 3 points meant social frailty. The Cronbach's alpha coefficient of the scale was 0.602 [14], and was 0.635 in the study.

#### The University of California at Los Angeles-Loneliness Scale (UCLA)

The University of California at Los Angeles (UCLA) -Loneliness Scale was developed by Russell et al. [21], with a total of 20 entries, using the Likert 4 rating scale (always = 4, sometimes = 3, rarely = 2, never = 1), of which 1, 5, 6, 9, 10, 15, 16, 19, 20 entries were scored reversely (always = 1, sometimes = 2, rarely = 3, never = 4). Higher scores meant the participants felt lonelier. There were three grades evaluated by the scores. “Not lonely” (scored 20–34 points), “somewhat lonely” (scored 35–49 points), and “lonely” (scored more than 50 points) [22]. The mean internal consistency reliability coefficient was 0.87 [23], and Cronbach's alpha coefficient was 0.885 in the Chinese population [24], and was 0.911 in this research.

### Statistical analyses

SPSS 21.0 and AMOS 23.0 were used for statistical analysis. The category data were expressed as frequency and percentage, and the continuous data were expressed as mean and standard deviation (SD). The scores of social frailty of the older adults in different characteristic communities were compared by independent sample t-test and ANOVA. The correlation between social frailty and loneliness was analysed by Pearson correlation analysis. The variables with statistical significance in the single factor analysis and the total score of loneliness were used as independent variables, and the multiple stepwise linear regression analysis was carried out with the score of social frailty as the dependent variable (the specific assignment of independent variables was shown in Table 1). Table 1 presented the assignment of the variables and the form of inclusion of each variable especially the hierarchical and dichotomous variables, which showed the results of our concern to some extent. The difference was statistically significant while* P* < 0.05. The structural equation modelling was conducted to estimate the fitness of the social frailty model, the model-fit indexes are as follows: relative chi-square (χ2/df) ≤ 3.00; root mean square error of approximation (RMSEA) ≤ 0.08 [25]; goodness of fit index (GFI), adjusted goodness of fit index (AGFI), Normed Fit Index (NFI), incremental fit index (IFI), Tucker-Lewis index (TLI) and comparative fit index (CFI) ≥ 0.9; standardized root mean square residual (SRMR) ≤ 0.05 [26]. The bootstrap was used 2000 times within the 95% confidence interval to test the significance of the direct, indirect, and total effects of the modified model.

**Table 1:** Assignment of independent variables

Variables	Assignment criteria
Age	60–74 = 1, 75–89 = 2, 90 or older = 3
Educational level	Primary and below = 1, Middle school = 2, Tertiary education = 3, Undergraduate and above = 4
Type of work	Mental labor = 1, Physical labor = 2, Both = 3
Marital status	Married(H1 = 0,H2 = 0,H3 = 0), Singlehood(H1 = 1,H2 = 0,H3 = 0) Widowed(H1 = 0,H2 = 1,H3 = 0), Divorce(H1 = 0,H2 = 0,H3 = 1)
Habitation	Solitude(J1 = 0,J2 = 0,J3 = 0), Living with spouse only(J1 = 1,J2 = 0,J3 = 0) Living with children and spouse(J1 = 0,J2 = 1,J3 = 0), Others ( such as pension institutions)(J1 = 0,J2 = 0,J3 = 1)
Family relations	Not harmony = 1, Normal harmony = 2, More harmonious = 3, Very harmonious = 4
Daily exercise	No = 0, Yes = 1
Sleep quality	Good = 1, Normal = 2, Poor = 3
Chronic disease	Less than 1 kind = 1, Greater than or equal to two = 2
Personal income	Income is greater than expenditure = 1, Income approximately equal to expenditure = 2, Income less than expenditure = 3, No income = 4
Medical burden	No burden = 1, Some burdens = 2, Burden heavier = 3
Total score of loneliness	Initial value

### Model modification

Initially, the SEM model of social frailty was built based on the social convoy model, but the index of model fitness was not really good. According to the literature review, there is a correlation between educational level and personal income [27, 28]. Meanwhile, financial difficulty (Q4) was related to personal income obviously. Finally, we adjusted the hypothesis model to combine the value of MI and literature review by adding the relationship between education and income, as well as income and e4.

## Results

### General data description of respondents

A total of 295 community-dwelling older adults were included, including 136 males (46.10%) and 158 females (53.90%); 176 cases aged 60–74 (59.66%), 105 cases aged 75–89 (35.59%), and 14 cases aged ≥ 90 (4.75%); the majority of primary and secondary school graduates (223/75.60%); most of them had spouses (239/81.02%) most of them lived with their spouses (175/59.32%) most of them had sports hobbies (171/57.97%); 102 cases (34.58%) of chronic diseases ≥ 2; 202 cases (68.47%) had medical burden (Table 2).

**Table 2:** Single factor analysis of social frailty of older adults (*n* = 295)

Characteristics	%/SD	n/mean	Social frailty		
			Score	t/F	*P*
Gender					
Male	46.10	136	2.13 ± 1.59	0.423	0.672
Female	53.90	159	2.06 ± 1.48		
Age					
60–74	59.66	176	1.88 ± 1.41^c^	7.544	**
75–89	35.59	105	2.28 ± 1.63^b^		
90 or older	4.75	14	3.36 ± 1.60^a^		
Educational level					
Primary and below	33.56	99	2.58 ± 1.59^a^	6.731	***
Middle school	42.03	124	1.91 ± 1.40^b^		
Tertiary education	14.24	42	2.02 ± 1.65^ab^		
Undergraduate and above	10.17	30	1.33 ± 1.21^b^		
Type of work					
Physical labor	43.39	128	2.31 ± 1.46^a^	3.198	*
Mental labor	28.47	84	1.77 ± 1.37^b^		
Both	28.14	83	2.07 ± 1.73^ab^		
Marital status					
Married	81.02	239	1.92 ± 1.46^c^	8.033	***
Singlehood	2.03	6	2.67 ± 1.86^abc^		
Widowed	14.58	43	2.58 ± 1.59^b^		
Divorce	2.37	7	4.29 ± 1.11^a^		
Habitation					
Solitude	11.86	35	2.91 ± 1.60^a^	7.266	***
Living with spouse only	59.32	175	1.84 ± 1.42^b^		
Living with children and spouse	25.08	74	2.14 ± 1.51^ab^		
Others	3.73	11	3.18 ± 1.94^a^		
Family relationship					
Not harmony	3.39	10	4.00 ± 1.70^a^	16.505	***
Normal harmony	22.37	66	2.65 ± 1.58^b^		
More harmony	43.39	128	2.14 ± 1.42^b^		
Very harmony	30.85	91	1.41 ± 1.27^c^		
Daily exercise					
Yes	57.97	171	1.78 ± 1.44	4.254	***
No	42.03	124	2.52 ± 1.55		
Sleep quality					
Good	42.37	125	1.66 ± 1.39^b^	11.979	***
Normal	43.39	128	2.27 ± 1.53^a^		
Poor	14.24	42	2.86 ± 1.54^a^		
Chronic disease					
Less than 1 kind	65.42	193	1.93 ± 1.41	-2.332	**
Greater than or equal to two	34.58	102	2.39 ± 1.71		
Personal income					
Income is greater than expenditure	29.15	86	1.38 ± 1.47^b^	11.630	***
Income approximately equal to expenditure	30.51	90	2.10 ± 1.32^a^		
Income less than expenditure	20.34	60	2.55 ± 1.50^a^		
No income	20.00	59	2.64 ± 1.58^a^		
Medical burden					
No burden	31.53	93	1.47 ± 1.43^c^	19.246	***
Some burdens	48.81	144	2.14 ± 1.38^b^		
Burden heavier	19.66	58	2.97 ± 1.61^a^		

The results of the post-hoc analysis (Bonferroni) were indicated by the letters a, b, and c in Table 1. Different letters indicated a statistical difference (*p* < 0.05), while the same letter indicated no significant difference between the two (*p* > 0.05)

^*^P ≤ 0.05; **P ≤ 0.01; ***P ≤ 0.001

### The status quo of social frailty and loneliness of the older adults in the community

The total score of the social frailty of the older adults in the community was (2.09 ± 1.53). It meant that the older adults in the community were in a state of pre-social frailty. In general, the total loneliness score of the community older adults was (43.19 ± 8.91), 74 cases (25.08%) had high loneliness, 155 cases (52.54%) had moderate loneliness, and 66 cases (22.37%) had low loneliness.

### Single-factor analysis of social frailty and loneliness among older adults in the community

The social frailty score differences in the older adults of different ages, education levels, marital status, type of work, habitation, family relationships, daily exercise, sleep quality, the number of chronic diseases, personal income, and medical burden were statistically significant (*P* < 0.05) (Table 2).

### Correlation analysis between social frailty and loneliness of the older adults in the community

The Pearson correlation coefficient between social frailty and loneliness was 0.621 (*P* < 0.01). Social frailty was moderately positively correlated with loneliness, and the Spearman correlation coefficient showed that all dimensions of social frailty were positively correlated with loneliness. See Table 3.

**Table 3:** Correlation between social frailty and loneliness of older adults (*n *= 295)

Characteristics	Aggregate score	
	*r*	*P*
Inability to help others	0.372	^***^
Limited social participation	0.467	***
Loneliness	0.410	***
Financial difficultly	0.405	***
Reduced social communication	0.393	***
Social frailty	0.621	***

^*^P ≤ 0.05; **P ≤ 0.01; ***P ≤ 0.001

### Multivariate analysis of the social frailty of older adults in the community

The results of multivariate analysis of the social frailty further clarified the influence of each variable on the social frailty of the older adults in the community, it showed that age, education level, living conditions, income and expenditure balance, and loneliness were the main influencing factors of the social frailty of older adults in the community (*P* < 0.05), which explained 49.5% of the total variation. See Table 4.

**Table 4:** Results of multiple stepwise linear regression analysis on influencing factors of social frailty of older adults (*n* = 295)

Variables	B	SE	SB	*t*	*P*
Constant value	-2.409	0.522		-4.614	***
Age	0.230	0.114	0.088	2.018	*
Educational level	-0.176	0.072	-0.109	-2.462	*
Habitation					
Living with spouse only	-0.581	0.267	-0.187	-2.176	*
Living with children and spouse	-0.467	0.253	-0.133	-1.849	0.066
Others (e.g., pension institutions)	-0.042	0.429	-0.005	-0.097	0.923
Personal income	0.218	0.063	0.156	3.459	***
Total score of loneliness	0.098	0.008	0.568	12.830	***

*F* = 27.180, *P* < 0.001; *R*^*2*^ = 0.514, adjusted *R*^*2*^ = 0.495

^*^P ≤ 0.05; **P ≤ 0.01; ***P ≤ 0.001

### Structural equation model of social frailty

**Fig. 2:**
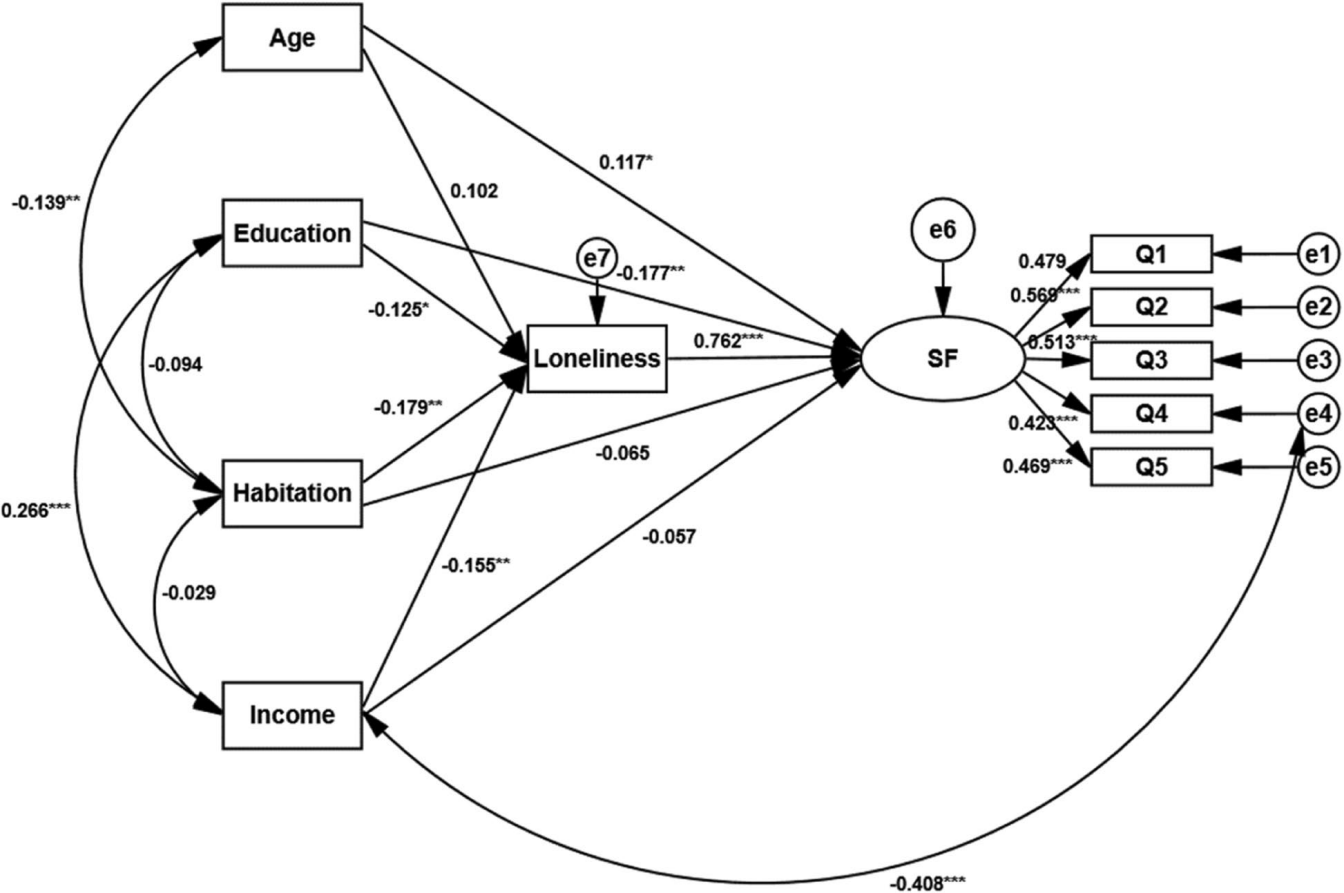
The standardized regression coefficients of structural equation models for social frailty *P ≤ 0.05; **P ≤ 0.01; ***P ≤ 0.001. Q1 ~ Q5: Five dimensions of social frailty; SF (Social frailty)

Based on the value of modification Indices, two correction lines were added between the variances to improve the fitness of the model (Fig. 2). The initial model indicated a satisfactory model fit (χ^2^ = 47.292, df = 26, χ^2^/df = 1.819, P = 0.007; RMSEA = 0.053, 95%CI (0.028, 0.076), *P* = 0.359; GFI = 0.971; AGFI = 0.939; NFI = 0.904; IFI = 0.955; TLI = 0.918; CFI = 0.953; SRMR = 0.0466). The results showed that age (β = 0.117, *P* = 0.037) and loneliness (β = 0.762, *P* < 0.001) had a positive effect on social frailty; Education level and had a negative effect on social frailty (β = -0.177, *P* = 0.003); The path from habitation (β = -0.065, *P* = 0.251) or income (β = -0.057, *P* = 0.346) to social frailty, together with the path from age to loneliness (β = 0.102, *P* = 0.069) were not significant (Fig. 2) (*P* > 0.05). Loneliness mediated the relationship between personal characteristics and social frailty (Tables 5 and 6).

**Table 5:** Path analysis of factors affecting social frailty (*n* = 295)

Path	Estimate	Standardized Estimate	S.E	C.R	*P*
Education–- > Loneliness	-1.174	-0.125	0.546	-2.152	*
Age–- > Loneliness	1.546	0.102	0.850	1.818	0.069
Habitation–- > Loneliness	-4.580	-0.179	1.444	-3.171	**
Income–- > Loneliness	-1.254	-0.155	0.466	-2.690	**
Loneliness–- > Social frailty	0.02	0.762	0.003	7.572	***
Education–- > Social frailty	-0.012	-0.057	0.013	-0.943	0.346
Habitation–- > Social frailty	-0.043	-0.065	0.038	-1.148	0.251
Age–- > Social frailty	0.046	0.117	0.022	2.089	*
Education–- > Social frailty	-0.044	-0.177	0.015	-2.93	**
Social frailty–- > Q1	1.000	0.479			
Social frailty–- > Q2	1.221	0.569	0.187	6.516	***
Social frailty–- > Q3	1.101	0.513	0.179	6.150	***
Social frailty –- > Q4	0.856	0.423	0.150	5.717	***
Social frailty –- > Q5	0.908	0.469	0.156	5.818	***
Habitation < – > Education	-0.031	-0.094	0.019	-1.619	0.105
Income < – > Habitation	-0.011	-0.029	0.020	-0.536	0.592
Age < – > Habitation	-0.028	-0.139	0.012	-2.369	**
Income < – > education	0.274	0.266	0.058	4.694	***
Income < – > e4	-0.183	-0.408	0.030	-6.173	***

Q1 ~ Q5: Five dimensions of social frailty

^*^P ≤ 0.05; **P ≤ 0.01; ***P ≤ 0.001

**Table 6:** Standardized effects of factors affecting in community-dwelling older adults with social frailty (*n* = 295)

Path	Direct effects	Indirect effects	Total effects
Age–- > Social frailty	0.117	0.078	0.195^*^
Income–- > Social frailty	-0.057	-0.118^**^	-0.175^*^
Habitation–- > Social frailty	-0.065	-0.136^**^	-0.201^**^
Education–- > Social frailty	-0.177^**^	-0.095^*^	-0.272^***^
Loneliness–- > Social frailty	0.762^***^	-	0.762^***^
Social frailty –- > Q1	0.469^***^	-	0.469^***^
Social frailty –- > Q2	0.423^***^	-	0.423^***^
Social frailty –- > Q3	0.513^***^	-	0.513^***^
Social frailty –- > Q4	0.569^***^	-	0.569^***^
Social frailty –- > Q5	0.479^***^	-	0.479^***^
Education–- > Loneliness	-0.125^*^	-	-0.125^*^
Income–- > Loneliness	0.155^**^	-	0.155^**^
Habitation–- > Loneliness	-0.179^**^	-	-0.179^**^
Age–- > Loneliness	0.102	-	0.102

Q1 ~ Q5: Five dimensions of social frailty

^*^P ≤ 0.05; **P ≤ 0.01; ***P ≤ 0.001

## Discussion

This cross-sectional study provided evidence for the relationship between loneliness and social frailty and explored the factors of social frailty in the older adults of the Chinese community. The study attached importance to the status of mental health and social frailty and emphasized the need for psycho-social interventions for older adults in the community. Based on the above study, structural models indicated that age, education level, and loneliness were the main predictors of social frailty among community-dwelling older adults. Age and education level had a direct effect on social frailty and indirect effects on social frailty through loneliness.

It was revealed that the status of older adults’ social frailty and loneliness was not optimistic. In this study, the prevalence of social frailty was 29.83%, and the proportion of the early stage of social frailty was 53.90%, which was significantly higher than a systematic overview of the prevalence of social frailty (22%) [29], which might be related to the different economic conditions of the selected provinces and cities and the number of older adults activities. Furthermore, the proportion of older adults with moderate or higher loneliness was 77.62%, slightly lower than that of the survey results of 5625 older adults in rural areas of AnHui (78.1%) made by Wang et al. [20]. This might be due to their observation of older adults in rural areas. Poor education, lack of regular income, lack of daily care, and the ability to accompany children were more likely to lead to loneliness among older adults in rural areas. Several studies had shown that the prevalence of loneliness was significantly higher in rural areas than in urban areas [30]. This difference might be closely related to factors such as the social environment, family structure, and economic level in rural areas [31]. Many populations in rural areas tended to face social isolation, aging, and poor mobility. Urban areas, on the other hand, were more likely to offer diverse social resources and a greater sense of attention and support from the surrounding environment. The loneliness levels of the older adults in the community were investigated by Susanty S et al. [32] and Lee S L et al. [33], both were lower than the results of this study. This may be based on the migration of adults from rural to urban areas, which is an important factor contributing to the relatively high prevalence of loneliness in urban areas [34]. As urbanization continues, there is an influx of rural people into cities, who often face multi-faceted adjustment problems in terms of culture, lifestyle, and social relationships are thus prone to negative feelings such as loneliness, helplessness, and loss. By contrast, residents of rural areas are more likely to enjoy the support of social networks such as fellow villagers and relatives, and are more likely to be influenced by the traditional culture of their communities [35]. In general, the prevalence of social frailty among the community older adults in China was high and showed an upward trend. The occurrence of loneliness was also not optimistic. Therefore, it is recommended that community workers pay more attention to and timely evaluate the social frailty and loneliness of older adults, enrich their daily activities, improve their social participation and play the role of social support. Once symptoms appear, effective intervention measures should be taken in time to improve the social frailty of older adults, prevent the occurrence of adverse health outcomes, and promote their physical and mental health.

In general, on the foundation of convoy model and results of previous multivariate analysis, our finding revealed the influencing factors of social frailty, as well as the relationship between social frailty and loneliness. Factor from individual characteristics included age, educational level and income. Similar to previous findings, higher levels of age among community-dwelling older adults were always accompanied by a higher incidence of social frailty. The reason may be that the degenerative changes in the body gradually increase with age. The decline of physical function makes older adults unable to effectively resist various adverse stimuli from the outside world, which is easy to lead to physiological weakness [36]. Some studies have shown that the existence of physiological weakness increases the risk of social frailty [37, 38]. It was also shown in the present study that the lower the educational level was, the higher the social frailty score was. The lower social participation of the older adults with low educational levels had no advantages in information acquisition and self-care. On the contrary, older adults with high educational levels reduced adverse psychological emotions by cultivating their interests and hobbies. Their self-worth and social participation were higher, and they had certain information acquisition abilities and self-care consciousness. Therefore, community workers should pay more attention to older adults with low educational levels and provide more health education and social support. In this study, the balance of income and expenditure was also an influencing factor of social frailty. Those with income less than expenditure or without income had a higher score of social frailty. The reason might be that the source of income of older adults after retirement was reduced, and with the increase of age, the expenditure on health care increased relatively [39]. The funds available for older adults could not meet their basic social needs, which was easy to cause social frailty of the older adults. As for the situational characteristics of social frailty was living condition. The scores of social frailty of the older adults living alone were higher than those of the older adults living with their spouses, which was consistent with previous studies [40, 41]. The results of previous studies had shown that sources of social support were classified as spouse, children living with their elders, children living apart, and friends/neighbors [42]. And all of the above factors can affect the loneliness of the elderly to varying degrees [43]. Because children were busy with work and had less companionship and communication with the older adults, the older adults mostly regarded their spouses as daily communication objects. However, the older adults living alone lacked corresponding partner support and emotional communication, so their loneliness increased, and the incidence of social frailty was higher [44, 45]. Therefore, timely attention should be paid to the mental and emotional state and social communication of older adults living alone.

Loneliness was an important positive predictor of social frailty (*β* = 0.762, *P* < 0.001). It was interesting to note that there was a moderate correlation between social frailty and loneliness of the older adults in the community (*r* = 0.621, *P* < 0.01). Loneliness was a significant indigenous influencing factor of social frailty. Loneliness meant a lack of emotional support from social support. The higher the loneliness was, the higher the score of social frailty was. When the older adults gradually entered the second half of their lives, they changed their social roles and reduced their social activities and communication to varying degrees. The shift in social relationships might reduce communication with people, which might further cause psychological stress to older adults [46]. Therefore, the loneliness experience of the older adults was more obvious. The generation of negative emotions such as loneliness affected the emotional regulation ability and participation in social activities of older adults, resulting in the decline of their physical function and life satisfaction, and the occurrence of social psychological crisis [47, 48]. At the same time, the results of this study suggested that all dimensions of social decline of the older adults in the community (social role, social participation, loneliness, economic level, and social communication) were positively correlated with loneliness, among which the social involvement dimension had the highest correlation. Studies have shown that older adults could greatly reduce their loneliness experience and improve their quality of life by actively participating in social activities and the process of social development [49–51]. This might be due to the older adults in the process of social participation, which could consolidate social networks, reduce social isolation, and enhance social integration, thereby inhibiting loneliness. In addition, studies have shown that team workouts, participation in collective activities, and social support interventions could have a significant impact on the mental health of older adults and help alleviate their social frailty of the older adults [52–54]. Therefore, community workers should realize the importance of social participation and support, encourage older adults to actively participate in social activities, reduce social frailty and loneliness, and effectively improve their quality of life. Communities could regularly conduct physical examinations for older adults and examine the situation of mental health and frailty, aiming for early detection and timely intervention [48].

It was interesting to note that, different from the existing studies [55], the present findings of the structural equation model suggested that the path from living conditions to social frailty and the path from income and expenditure balance to social frailty were not significant. The living styles and income and expenditure balance could not act on the social frailty of the older adults in the community. It may be related to the small proportion of older adults living alone in this study. In the future, it was necessary to further explore the relationship between living styles and social frailty. When it came to the income and expenditure balance, it might be the older adults themselves have economic deposits or economic support provided by their children, and the balance of income and expenditure didn’t work on the social frailty of the older adults. In conclusion, community medical workers should pay attention to the mental health status of older adults on time, reduce the loneliness experience of older adults and reduce the occurrence of social frailty from multiple perspectives, such as social participation, social support, and social communication.

The investigate of the relationship of social frailty and loneliness has positive implications for the promotion of healthy aging. Healthy Ageing is the process of developing and maintaining the functional ability that enables well-being in older age [56], which mean a person’s ability to build and maintain relationships also contribute to the healthy aging [57]. The SEM model of social frailty provides guidance that intervention programs of social frailty in in community-dwelling older adults can take into account the characteristics of older adults, especially the older or those with lower levels of education. Meanwhile, taking some measures to reduce loneliness among older persons can also contribute to the decrease in social frailty, and thus enhance the social connections of older adults. Indeed, it has a positive impact on healthy ageing in terms of maintaining social relationships.

### Limitations

The limitation of this study was that only a cross-sectional study could not verify the causal relationship between social frailty and loneliness. Besides, the study employed convenience sampling and snowball sampling methods, which might limit the representative of the sample and generalization of the results. Further, the process of data collection utilised self-administered and assisted support, which might affect the accuracy and uniformity of answers, as some participants might have received different levels of guidance or instructions. Moreover, the study's participants were all drawn from a single geographic location, which might limit the generalisability of the findings to other areas. Follow-up researchers can conduct longitudinal studies to accumulate evidence for the causal relationship between social frailty and loneliness, focus on the impact of social frailty and loneliness on the health status of older adults and explore effective interventions.

## Conclusions

Based on convoy model, the structural equation model of factors affecting older adults with social frailty living in the community was conducted. Age, education level, and loneliness were the main predictors of social frailty among community-dwelling older adults. The incidence of social frailty among the community older adults in China was high, and loneliness was at a medium level. There was a positive correlation between social frailty and loneliness. Under the background of global advocacy for healthy aging, community medical workers should pay more attention to the social frailty of older adults. According to the differences in age and education level of older adults, personalized health intervention plans should be implemented. Social frailty intervention programs should be categorically designed and implemented. The intervention measures will play a role in social frailty to the greatest extent, improve the physical and mental health of older adults, and promote the development of healthy aging.

## Data Availability

The datasets used and/or analyzed during the current study are available from the corresponding author upon reasonable request.

## Abbreviations

SF: Social Frailty
UCLA: The University of California at Los Angeles-Loneliness Scale
SD: Standard deviation
HALFT: Help, Participation, Loneliness, Financial, Talk Scale
